# Reflective practice in health care and how to reflect effectively

**DOI:** 10.1097/IJ9.0000000000000020

**Published:** 2017-06-15

**Authors:** Kiron Koshy, Christopher Limb, Buket Gundogan, Katharine Whitehurst, Daniyal J. Jafree

**Affiliations:** a Brighton and Sussex University Hospital; b Western Sussex University Hospitals, Worthing; c UCL Medical School, University College London, London; d Royal Devon and Exeter Hospital, UK

**Keywords:** Reflection, Reflective practice, Portfolio, Career progression, Learning

## Abstract

Reflective practice is a paper requirement of your career progression in health care. However, if done properly, it can greatly improve your skills as a health care provider. This article provides some structure to reflective practice to allow a health care provider to engage more with reflective practice and get more out of the experience.

## Introduction

Reflective practice is something most people first formally encounter at university. This may be reflecting on a patient case, or an elective, or other experience. However, what you may not have considered is that you have been subconsciously reflecting your whole life: thinking about and learning from past experiences to avoid things that did not work and to repeat things that did. For example after tasting a food you do not like, you remember that experience, think about it, and when you next see that same food you know to avoid it. In medicine it is one of the best approaches to convert theoretical knowledge into practice.

As you progress through medical school and into foundation years as a doctor it becomes even more common. It is now expected to provide evidence of your reflections through your training on the ePortfolio and then throughout the rest of your professional life in revalidation. Hence, it is a good idea to get it right from the beginning.

First and foremost the biggest mistake you can make when reflecting is to treat it as a tick box exercise and a waste of time. With a bit of thought reflections can be a very useful tool in learning. Would you remember a generic case from a book? Would hanging all of those facts on a patient you have met make it more memorable? It allows you to recognize your own strengths and weakness, and use this to guide on-going learning. By reflection you will develop your skills in self-directed learning, improve motivation, and improve the quality of care you are able to provide.

## What to reflect on

This can be anything.

Most reflections are on things that go wrong. These situations stay in one’s head and force us to begin to think about whether they could have done anything differently. For example:

- Postoperative complications
- Missed diagnosis
- A dissatisfied patient
- Failed procedure

However, reflecting on things that went well can often be more rewarding and be just as useful. It can build confidence and help you to repeat it again on another occasion. For example:

- A well-managed cardiac arrest
- An interesting seminar or conference
- A patient thank you letter
- A difficult but well performed procedure

## Stages of reflecting

There are numerous models for reflections, but it is important to understand why you are asking each question and how that will help you to reflect^1^. This an integration of many concepts but the broad process is similar in all models: what happened, why does this matter and what are the next steps?^2^

### What, where, and who—the situation

Think about the situation in detail: What happened exactly and in what order, where were you at the time and who else was involved? What part did you have to play? What was the final outcome?

### How did it make you feel—your emotional state

What was running through your head and how did you feel about it? Be honest with yourself: were you afraid, confused, angry or scared? If you can understand how you were feeling at the time it will help you put together why things happened as they did, and help you to recognize similar situations in the future.

### Why did it happen—making sense of the situation

Now you have thought about the situation in greater detail, and probably recognized things that would have otherwise gone unnoticed, think about why things happened as they did. How did the situation, yourself, and others interact at the time. Did the situation go well or was there room for improvement?

### Could you have done anything differently—critical review and development of insight

With the help of hindsight how would you have managed the situation differently? Think about what factors you could have influenced: is there anything you could have tried that may have improved the situation, or is there anything you did that was particularly important in the situation? It is easy to remember the things that you did not do and it is often the things that you did well that are forgotten.

### What will you do differently in the future—how will this change your practice

This is arguably the most important stage in reflecting. You need to pull together everything you have thought of before to learn, change your own practice, and improve^3^. Do not only think about what you would do differently in that specific situation, but think whether you have thought of any transferable knowledge or skills you can utilize elsewhere. For example: if you reflect on a postprocedural complication do not only think of how you would manage this again but also how you would prevent it happening if you performed the procedure yourself! If you are a part of a well-led cardiac arrest do not think only of what you would do next to help, but also how you would lead an arrest in the future, or even how you would lead a team in any other situation!

### Re-enforcement—what happens when you put this into practice

Test your reflections: When comparable situations happen again, do things change as you would expect them to? This is a chance to repeat the reflective cycle to refine and develop your understanding.

## How to make the best use of reflective practice

As mentioned previously most people see reflective practice as a tick box exercise, but it does not have to be.

Over the next day take note of any interesting situations that arise. Later in the day try mentally reflecting, following this framework, and if you think any will be particularly useful to you write them down. If you try this for a week you will begin to see similar situations arising and how your reflective practice is positively affecting you.

Remember: you do not always have to learn only from your own experience; learn from others’ mistakes as well. Reflect on situations that you have witnessed to work out why things happened as they did, and how this can influence you.

It can be useful to take these reflections for peer or senior review: others may be able to draw light on things you have not noticed. This can allow you to recognize points for improvement and work on them. This can also be a useful learning opportunity for the other involved!

## An example to put this into practice

### Bad

I was involved in a patient confrontation; the patient was unhappy with her hospital stay and wanted to be discharged home. Unfortunately she required a package of care and so could not be discharged. I explained this and she returned to her bed. I was happy I had explained everything to her and continued with my other jobs.

### Good

#### Who, what, and why

I was involved in a patient confrontation; an elderly patient was unhappy with hospital stay and wanted to be discharged home. She was under our general surgical team for a head injury and observation after a normal CT head. She had been seen on our ward round and told that she was medically fit for discharge but still awaiting social services: her house had been reviewed and deemed unsafe so she was waiting for banisters to be installed. The issue was raised with me by chance as I was doing other things on the ward. I explained this to her and although she remained annoyed I was able to make her understand what the delay was and she returned to her bedside. She did not seek further clarification that day.

#### How did it make you feel

At the time I felt rushed and frustrated. I had a lot of other work to be done and this was distracting from that. She had already been told she was waiting for social services in the morning. I understood why this was difficult for her but did not think I would be able to do anything to help.

#### Why did it happen

The morning ward round was quite rushed and so our explanation was limited to telling her we were waiting for social services. I can understand from her point of view this may have meant very little, and so my explanation of what exactly we were doing may have relieved some frustration. Having been waiting up to this point, it is no surprise she continued to be angry but may have been accepting of this plan.

#### Could you have done anything differently

I think my explanation was very good, and the patient seemed happy with this, although I did not give a rough idea of how long this would take. It may have been useful to have spoken to the sister in charge to ask for what progress had been made to feed back to the patient. Also I did not ask her whether she was happy with this explanation: I may have been able to satisfy her frustration further by answering a few more questions or even recognize any other issues at home that may need addressing before discharge. Although the information given in the ward round was correct, it was not understandable to the patient. If this had all been quickly clarified in the morning, the patient would have been happy throughout the day and not caused a problem later on.

#### What will you do differently in the future

I think that the route problem in this situation was our explanation on the morning ward round. Furthermore, I am not sure how long such issues take to be addressed. To avoid a similar situation in the future I will speak to the other health care professionals on the ward to get a round idea of how long occupational interventions such as this and other community interventions take to start. This means when future patients are medically fit I can spend a moment in the morning informing them of what needs to be done and how long it may take. Hopefully this will allow me to address patient concerns early to avoid them becoming an issue when it is too late.

#### Re-enforcement

I will reflect on how future situations similar to this develop, looking for an improvement in the quality of my patient care.

Following a structure helps to focus a reflection: I am sure you will agree the learning points are much clearer from a good reflection!

## Conclusions

To summarize, the benefits of reflecting are clear: it may be difficult to do initially, but through practice you will develop your own skills and become a better learner. Many structures are available so choose one what works for you. Reflective practice is an important part of your career progression on paper, but if done well, can greatly improve your skills as a health care provider.

## References

[1] Gibbs G Learning by Doing: A Guide to Teaching and Learning Methods. Oxford: Further Educational Unit, Oxford Polytechnic; 1988.

[2] Rolfe G Freshwater D Jasper M Critical Reflection in Nursing and the Helping Professions: a User’s Guide. Basingstoke: Palgrave Macmillan; 2001.

[3] Schön DA The Reflective Practitioner: How Professionals Think in Action. New York, NY: Basic Books; 1983.

